# Public Acceptability in the UK and USA of Nudging to Reduce Obesity: The Example of Reducing Sugar-Sweetened Beverages Consumption

**DOI:** 10.1371/journal.pone.0155995

**Published:** 2016-06-08

**Authors:** Dragos C. Petrescu, Gareth J. Hollands, Dominique-Laurent Couturier, Yin-Lam Ng, Theresa M. Marteau

**Affiliations:** Behaviour and Health Research Unit, Institute of Public Health, University of Cambridge, Cambridge, United Kingdom; Indiana University, UNITED STATES

## Abstract

**Background:**

“Nudging”—modifying environments to change people’s behavior, often without their conscious awareness—can improve health, but public acceptability of nudging is largely unknown.

**Methods:**

We compared acceptability, in the United Kingdom (UK) and the United States of America (USA), of government interventions to reduce consumption of sugar-sweetened beverages. Three nudge interventions were assessed: i. reducing portion Size, ii. changing the Shape of the drink containers, iii. changing their shelf Location; alongside two traditional interventions: iv. Taxation and v. Education. We also tested the hypothesis that describing interventions as working through non-conscious processes decreases their acceptability. Predictors of acceptability, including perceived intervention effectiveness, were also assessed. Participants (n = 1093 UK and n = 1082 USA) received a description of each of the five interventions which varied, by randomisation, in how the interventions were said to affect behaviour: (a) via conscious processes; (b) via non-conscious processes; or (c) no process stated. Acceptability was derived from responses to three items.

**Results:**

Levels of acceptability for four of the five interventions did not differ significantly between the UK and US samples; reducing portion size was less accepted by the US sample. Within each country, Education was rated as most acceptable and Taxation the least, with the three nudge-type interventions rated between these. There was no evidence to support the study hypothesis: i.e. stating that interventions worked via non-conscious processes did not decrease their acceptability in either the UK or US samples. Perceived effectiveness was the strongest predictor of acceptability for all interventions across the two samples.

**Conclusion:**

In conclusion, nudge interventions to reduce consumption of sugar-sweetened beverages seem similarly acceptable in the UK and USA, being more acceptable than taxation, but less acceptable than education. Contrary to prediction, we found no evidence that highlighting the non-conscious processes by which nudge interventions may work decreases their acceptability. However, highlighting the effectiveness of all interventions has the potential to increase their acceptability.

## Introduction

Much of the global health burden of chronic diseases is associated with modifiable behaviors, principally smoking, alcohol consumption, physical inactivity, and unhealthy food consumption [1]. Altering the environments in which behavior occurs—the “choice architecture”, also known as “nudging”—has the potential to change behavior in populations [2–6]. Whether such interventions are implemented depends not only on their effectiveness, but also on their acceptability to policymakers, a judgement likely influenced by perceived public acceptability. The focus of this study is upon the latter.

Public acceptability of nudge interventions to improve population health is largely unknown. Responses to a recent attempt to nudge populations to improve health entailed altering the sizes of sugar-sweetened beverages to reduce the amount of consumption. New York City Mayor Michael Bloomberg proposed limiting the size at which sugar-sweetened beverages could be sold in restaurants and other venues serving food [7, 8]. This proposal elicited strong negative views, as evidenced by the results of a public opinion poll suggesting that 60% of New Yorkers opposed such a measure [9]. It is possible that these views may have been influenced by campaigns ran by industry-funded consumer groups that placed advertisements on billboards and in newspapers which asserted that this measure negates individual freedom. Other concerns have been raised by ethicists largely focused upon the ability of nudge interventions to circumvent the need for deliberation prior to action [10]. Some ethicists have argued that this circumvention renders such interventions coercive [11].

The present study was designed to address three research questions regarding the public acceptability of nudging to improve population health, each outlined below. We used the example of interventions to reduce obesity, focusing specifically on interventions aimed at reducing the consumption of sugar-sweetened beverages.

### Research question 1: How acceptable are different types of interventions?

The primary aim of the present study was to describe public acceptability of a range of “nudge” and more traditional interventions. We assessed levels of public support for five interventions that could reduce consumption of sugar-sweetened beverages, three of which involved “nudging”: i. reducing portion Size, ii. changing the Shape of the drink containers and iii. changing their shelf Location. The acceptability of two other, more traditional types of intervention were also assessed: iv. Taxation and v. Education.

A recent systematic review revealed that acceptability of interventions to change health-related behaviors varies with their level of intrusiveness, with greater support evident for the provision of information, and less support evident for the use of financial disincentives such as taxation [12]. Just under half of the 200 studies included in this review were from the USA. The current study is conducted using UK and USA participants to ascertain the extent to which acceptability of interventions differ between these countries. The UK and the USA are two countries in which nudge interventions are being actively considered and sometimes implemented by policy-makers [13]. These two countries do, however, differ in their views on the use of prohibitive legislation to change behavior, with populations in the USA generally less supportive than those in the UK. For example, in a recent survey, only one third of American respondents supported more restrictive policies to address health-related concerns relating to diet and smoking, in contrast to almost half of the British sample that supported these policies [14]. It is unknown whether such differences are evident in attitudes towards other interventions.

### Research question 2: What is the impact on acceptability of highlighting conscious vs. non-conscious mechanisms?

Concerns regarding nudge interventions have focused on the non-conscious processes that likely mediate their impact [10, 11, 15]. As detailed earlier, ethicists as well as others, have argued that nudges manipulate decision-making processes, and may therefore be coercive. White [11] states “…*and*, *to make matters worse*, *the coercion operates through the very cognitive biases that behavioral economists use to justify the interventions*” (p.91). Concerns such as those voiced by White are particularly pertinent in the context of the public acceptability of nudge interventions because these interventions are likely to affect behavior via non-conscious processes [4, 6]. They are therefore likely to be presented or understood by the public in these terms. To our knowledge just one study, conducted by Felsen, Castelo & Reiner (2013), has assessed the impact on acceptability of the extent to which an intervention is mediated by conscious processes; finding higher acceptability for interventions presented as operating via conscious processes [16]. From the design of this study (involving just two intervention groups and no control group) it is unclear whether participants were responding negatively to the mention of subconscious processes, positively to the mention of conscious processes, or a combination of the two. A second aim of our study was to replicate this study, and in particular to distinguish between these different possible effects by adding a control group that did not specify whether the effect is elicited via conscious or non-conscious processes.

### Research question 3: What predicts acceptability?

The third aim of the present study was to explore the predictors of public acceptability of nudging. To date, studies that have assessed public acceptability of government interventions to improve population health reveal several predictors. These include: perceived effectiveness of each intervention, with acceptability increasing with effectiveness [17]; the belief that lack of willpower is responsible for overweight [18]; the belief that the environment is responsible for overweight [18]; trust in government [14]; political orientation [19]; perceived need for help in making healthier choices [16], and body-mass index (BMI) [20]. Moreover, the belief that human behavior is largely intentional (dispositionism) and the belief that behavior is largely shaped by the environment (situationism [21]) may also predict public attitudes to interventions. In the current study we therefore included measures of these constructs as potential predictors of support for nudge and more traditional interventions.

## Materials and Methods

### Study design

The study comprised a between-subjects design with participants randomly assigned to one of three groups. These groups varied in the information participants were given about the mechanism by which each of the five interventions were stated to affect behavior: (a) via conscious processes; (b) via non-conscious processes; (c) no explicit cognitive process stated. Data from the two countries were collected and analysed independently.

### Samples and recruitment

Both the UK participants (England only; *n* = 1093) and the USA participants (*n* = 1082) completed the study online.

#### UK sample

Recruitment was completed by the market research company ‘Survey Sampling International’ [SSI; http://www.surveysampling.co.uk/]. Participants were invited to take part in the study either by email, or when they visited a website where SSI listed current studies. Recruitment quotas were set so that the sample included similar numbers of participants by gender, and social grade (a system of socio-economic classification based on occupation, widely used in the UK). With regard to age and location, we requested that the recruited sample include a wide range of participants.

#### USA sample

Recruitment was conducted using Amazon Mechanical Turk [MT; https://www.mturk.com/mturk/welcome], the sampling frame used by Felsen et al.,[16] in their comparable study. Members of the MT panel were invited to complete the study online by accessing an advertised link. The study was only available to USA-based participants aged 18 and above. No other recruitment criteria were used.

The sample sizes were calculated based on data from Felsen et al.,[16]. The original study [16] used a between-subjects design and included a non-nationally representative sample of Americans (90.3%) and Canadians (9.7%; Gidon Felsen, PhD, email communication, December 10, 2013). The dependent measure was assessed on a 9-point scale and differences between groups (when effects were seen) were between 0.48 and 1.08. The observed standard deviations were in the range 1.41 to 2.42. Taking a conservative approach using the smallest effect size and the largest standard deviation seen (0.5 and 2.42 respectively) we would expect to find a standardised effect size of *d =* 0.21. The sample size required for 80% power to find this effect was 356 per group (2-sided significance of 0.05). Across all three groups the total sample size required was 1068 within each country.

### Measures

#### Primary outcome measure

**Fig 1:**
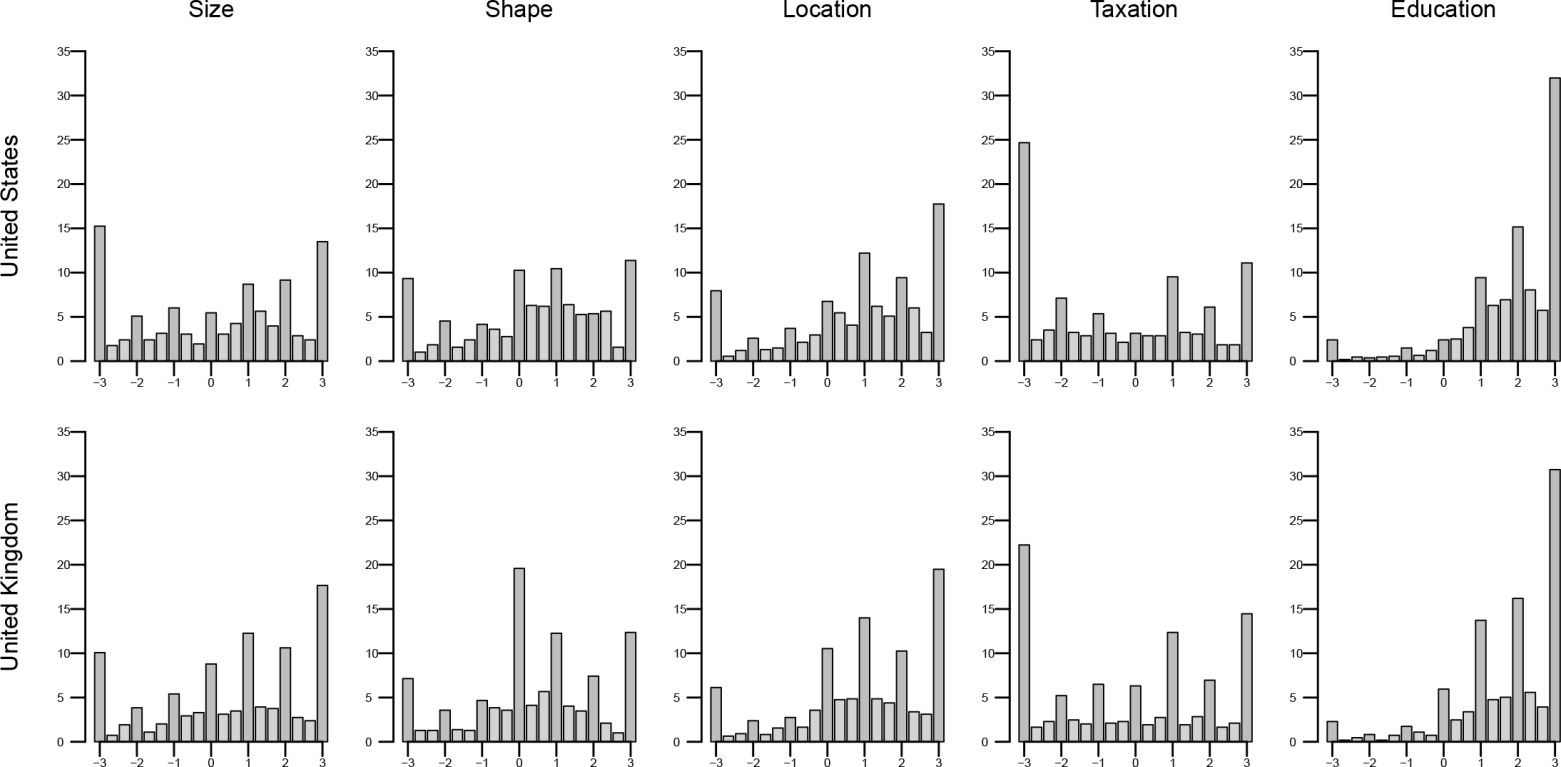
Barplots of the overall acceptability scores per intervention and country.

Acceptability of each intervention was assessed from ratings on three items anchored with values ranging from -3 to +3: i. “Do you support or oppose this policy?”, ii. “How acceptable do you find this policy?”, and iii. “How much are you in favour of this policy being introduced?” Overall acceptability scores were obtained using mean responses on these items (Cronbach's *α* values ranged between .96 and .99, for the five interventions across the two samples). Given the distribution of the data (Fig 1), responses were classified as denoting either acceptability (mean scores on the three items greater than zero) or not showing acceptability (mean scores on the three items at zero or lower). Dichotomising outcome variables is not uncommon practice in studies of public attitudes towards government interventions to improve population health in relation to obesity [22, 23], tobacco smoking [24] and alcohol consumption [25].

#### Independent predictors of acceptability

Perceived effectiveness of each intervention was assessed after the presentation of the intervention, using a single item rating on a scale from -3 to +3. Additional independent predictors of acceptability were assessed with similar self-report measures: situationism, dispositionism, trust in government [14], political orientation [19], beliefs regarding the causes for overweight [18], perceived need for help in making healthier choices [16] (see Measures for predictors of acceptability in S1 Appendix for actual items).

Demographic variables of age, gender, BMI, and socio-economic status (determined by highest educational qualification and social grade (occupational group) for the UK, and highest qualification and income for the USA) were also assessed. In addition, in line with recent concerns regarding participants’ attention in unsupervised settings [26], two quality-check items were included (see Measures for predictors of acceptability in S1 Appendix for actual items).

### Procedure

The study protocol was approved by the Cambridge Psychology Research Ethics Committee (Pre. 2013.86). Participants completed the study online and were randomly allocated to experimental conditions using the algorithm embedded in Qualtrics (the software used to program the task (http://www.qualtrics.com/)).

Participants were presented with an introductory text regarding obesity and sugar-sweetened beverages and a brief description of the five proposed government interventions, the order of the latter determined by randomisation. Participants in the non-conscious condition were informed that interventions affect behavior via non-conscious processes whereas participants in the conscious condition were informed that the interventions affect behavior via conscious processes. Participants in the control condition were not provided with any information regarding the mechanisms through which interventions were expected to work (see Box A in S2 Appendix), for the wording for all three study groups and each of the five interventions).

Following the description of each intervention, participants completed acceptability and effectiveness ratings. Finally, participants completed the remaining measures, listed above. At the end of the study participants were thanked and debriefed.

### Data analysis

#### Research question 1

To describe the acceptability level for each intervention, within and between each country, we did the following: (i) tested to see if the proportions of participants classified as accepting each intervention (overall acceptability score larger than zero) was significantly different from 50% within each country; (ii) analysed the difference in the proportions of participants classified as accepting each combination of interventions within each country and (iii) compared the proportions of participants judging as acceptable each intervention between the USA and the UK.

As our samples were not nationally representative (see Results for details), we weighted the data using UK and USA census data to generate percentage estimates allowing us to draw conclusions that reflect nationally representative samples. Weights were computed using the Iterative Proportional Fitting (IPF) algorithm [27, 28]. The chosen IPF population margins for the UK were: (1) `age groups’ ([18;30[, [30;40[, [40;50[, [50;65[, [65;Inf[) crossed with `gender’, and (2) `SES groups’ (DE, C1C2, AB), and for the US (1) age groups’ ([18;25[, [25;30[, [30;40[, [40;Inf[) crossed with `gender’, and (2) `household income groups’ ([0;25K[, [25K,50K[, [50K;Inf[). The age and household income groups were chosen so that the sample size per intersection of the chosen margins would be large enough for the IPF to run smoothly.

Inference for weighted estimators is complex [29, 30], we therefore used bootstrapping to define confidence intervals, as this method allows the variability of the weights to be taken into account, which is essential. We followed the approach Canty and Davison (1999)[31] used in a similar case involving a complex stratified survey, and IPF weights. We performed 35 non-parametric tests (*In total*, *35 CIs were calculated as follows*: *10 CIs for the percentage of acceptability of each intervention (5) within each country (2)*, *20 CIs for the differences of acceptability of each pair of interventions (10) within each country (2)*, *5 CIs for the differences in acceptability of each intervention between the two countries (5*)), in comparing levels of acceptability between interventions, within and between countries, correcting the type I error of each test using an improved version of the Bonferroni correction taking into account the dependence between the parameters of interest to achieve a 5% familywise error rate. Bretz et al., 2011 [32] (Sections 3.2 and 5.1) provide an in-depth description of the method, which requires an estimate of the correlation between the parameters of interest which we obtained by means of the non-parametric bootstrap described above. As a result, we analysed CIs with a coverage level of 0.99807. We opted for percentile bootstrap CIs[33] and a high number of simulated samples (25,000) due to the high chosen coverage level. Unweighted confidence intervals are reported for comparison purposes, but conclusions are drawn based on the weighted confidence intervals.

#### Research questions 2 and 3

To test for the impact of experimental condition, as well as the degree to which other independent variables predicted acceptability over and above the experimental manipulation, we conducted logistic regressions in which the probability of accepting an intervention (overall acceptability score larger than zero) was linked to predictors through the logit link function. Logistic models were estimated by means of iterative reweighted least squares

(IWLS). We used unweighted regressions including in our models all the variables that were used in the IPF described in the previous section as well as all their interactions [29, 30, 34].

Our choice of models was supported by diagnostic tests including the analysis of normalized randomized quantile residuals[35], sensitivity analyses to outliers by means of robust generalized linear models [36], and to the impact of multicollinearity.

Predictors were entered into the model in two steps: experimental condition together with control variables and their interactions were entered first to address Research question 2, and other predictors were included in Step 2 to address Research question 3.

#### UK sample

The experimental condition was entered in Step 1 with the control condition taken as reference. The demographic control variables characteristics were also entered in Step 1 as follows: gender (men taken as reference), age ([18; 30[taken as reference), education (the lowest level of education taken as reference), occupational group (group “DE” taken as reference), and ethnic group (White vs. Non-White, with the former taken as reference). Participants’ beliefs and other characteristics were entered into the model in Step 2, as standardised continuous independent variables: political orientation, trust in government, situationism, dispositionism, the two items pertaining to beliefs about overweight causes, the degree to which participants thought they needed help in making healthier food choices, and perceived effectiveness of the respective intervention.

#### USA sample

Equivalent steps were performed to predict acceptability of interventions in the USA sample. The differences concerned two variables assessing socio-economic status entered in Step 2: household income ([0; 25K[), and education entered as a categorical variable (lowest level, high school or less, were taken as reference).

For Research question 2, we used again a multiplicity correction taking into account the dependence between the parameters of interest to achieve a 5% familywise error rate, as we analysed the significance of 20 correlated parameters (five interventions, two countries, two consciousness dummies). Similar to Research question 1, we defined the correlation matrix of the contrasts of interest by means of a non-parametric bootstrap respecting the characteristics of our samples. As a result, we use 0.0027358 as cutting value for the p-values of the regression coefficients of interests. Research question 3 is exploratory, therefore, we chose 0.005 as the cut-off point for *p* values which corresponds to a Bonferroni correction for 10 outcomes (i.e., five interventions times two countries).

## Results

Characteristics of participants in the study are shown in Table 1.

**Table 1:** Demographic characteristics of the UK and USA study samples.

UK Sample (n = 1093)		USA Sample (n = 1082)	
**Gender**		**Gender**	
Men, % (n)	49.3 (539)	Men, % (n)	53.9 (583)
Women, % (n)	50.7 (554)	Women, % (n)	46.1 (499)
**Age**		**Age**	
18–35, % (n)	24.1 (263)	18–35, % (n)	68.9 (746)
36–65, % (n)	58.2 (636)	36–65, % (n)	29.9 (323)
66+, % (n)	17.7 (194)	66+, % (n)	1.2 (13)
**SES-Social grade**		**SES-Income**	
DE, % (n)	33.5 (366)	< $50000, % (n)	59.9 (648)
C1C2, % (n)	33.3 (364)	> $50000, % (n)	39.9 (432)
AB, % (n)	33.2 (363)	Missing, % (n)	.2 (2)
**SES-Education**^**ɨ**^		**SES-Education**	
Low, % (n)	20.4 (223)	High school or less, % (n)	13.9 (150)
Medium-low, % (n)	20.7 (226)	Some college, % (n)	34.8 (376)
Medium-high, % (n)	17.1 (187)	Degree or above, % (n)	51.4 (556)
High, % (n)	39.3 (430)		
Missing, % (n)	2.5 (27)		

^**ɨ**^N.B.: Low = Fewer than 5 GCSEs or equivalent; Medium-low: 5+ GCSEs/1 A level or equivalent; Medium high: 2+ A levels or equivalent; High: university degree or above.

As stated above, the samples differed from nationally representative samples. The UK sample was similar in terms of gender (51.3% of those aged 18 and over in England are women) and age (the mean age of the English population is 39.7 years). With regard to social grade the sample included approximately equal proportions of participants from each occupational group whereas a nationally representative sample would include mostly participants from mid grades (C1, C2 groups) (51.55%). The US sample differed from national representativeness in terms of gender (46.1% vs. 50.8% women), age (almost 70% being younger than 35 years of age, which is younger than the mean age of the US population (37.6 years)) and income (59.9% in the current sample have a total household income < $50000 vs 48% at a national level), reflecting the younger age of the sample.

### Research question 1: How acceptable are different types of interventions?

**Fig 2:**
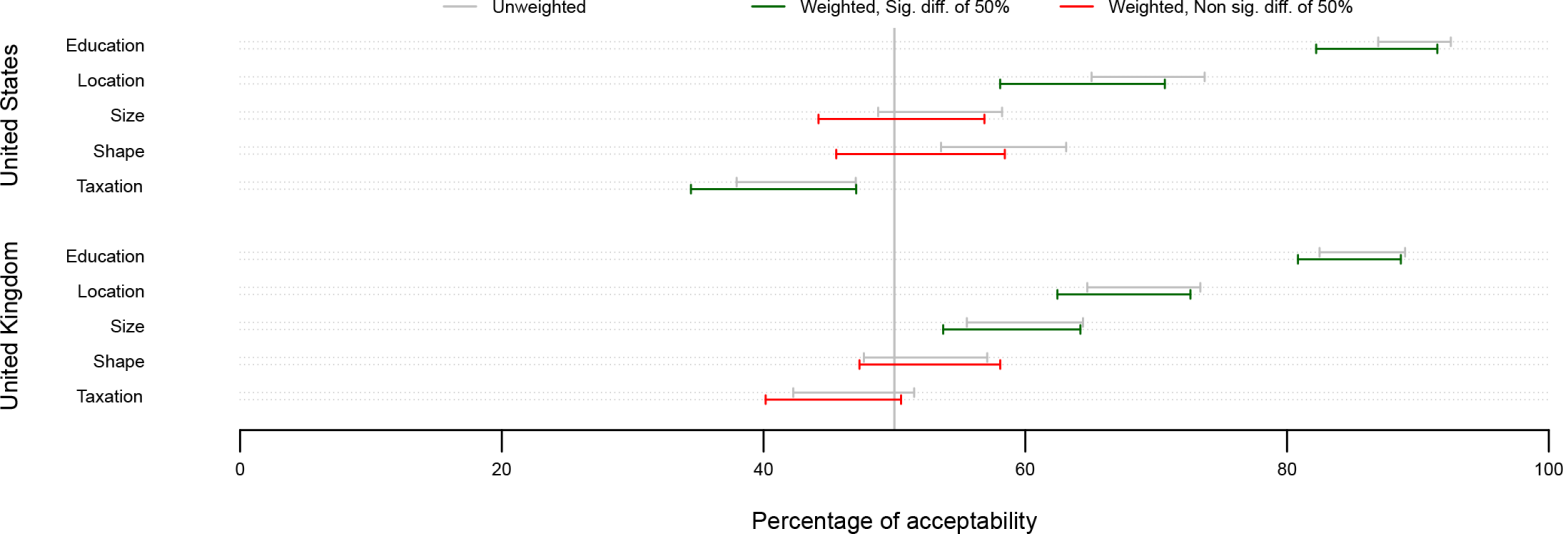
Proportion of participants (99.807% bootstrap percentile CIs) rating as acceptable each intervention, by country. Percentages significantly different from 50% appear in green. Percentages not significantly different from 50% appear in red. As 35 comparisons are performed in the analysis corresponding to Figs 2, 3 and 4, a correction for multiple testing which takes into account the dependencies in the data was used, leading to CI levels of 99.807%.

The proportions of participants judging each intervention acceptable by country, using unweighted and weighted data, are shown in Tables 2 and 3 and Fig 2.

**Table 2:** Weighted Percentage of participants (% (n)) rating as acceptable; [99.807% bootstrap percentile CIs] each intervention, by country.

	UK Sample (n = 1093)	USA Sample (n = 1082)
**Intervention**		
Education	84.9% (938) [80.8;88.7]	87.0% (972) [82.2;91.5]
Location	67.6% (755) [62.5;72.6]	64.4% (751) [58.1;70.7]
Size	59.0% (655) [53.7;64.2]	50.6% (579) [44.2;56.9]
Shape	52.7% (573) [47.3;58.1]	51.9% (633) [45.6;58.4]
Taxation	45.4% (513) [40.2;50.5]	40.7% (459) [34.5;47.1]

**Fig 3:**
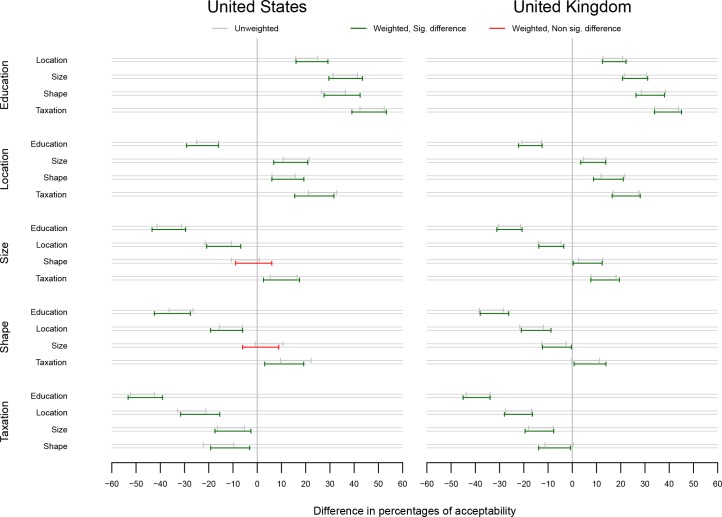
Difference in acceptability (99.807% bootstrap percentile CIs) for each combination of intervention within each country. Percentages significantly different from 0 appear in green. Percentages not significantly different from 0 appear in red. As 35 comparisons are performed in the analysis corresponding to Figs 2, 3 and 4, a correction for multiple testing which takes into account the dependencies in the data was used, leading to CI levels of 99.807%.

**Fig 4:**
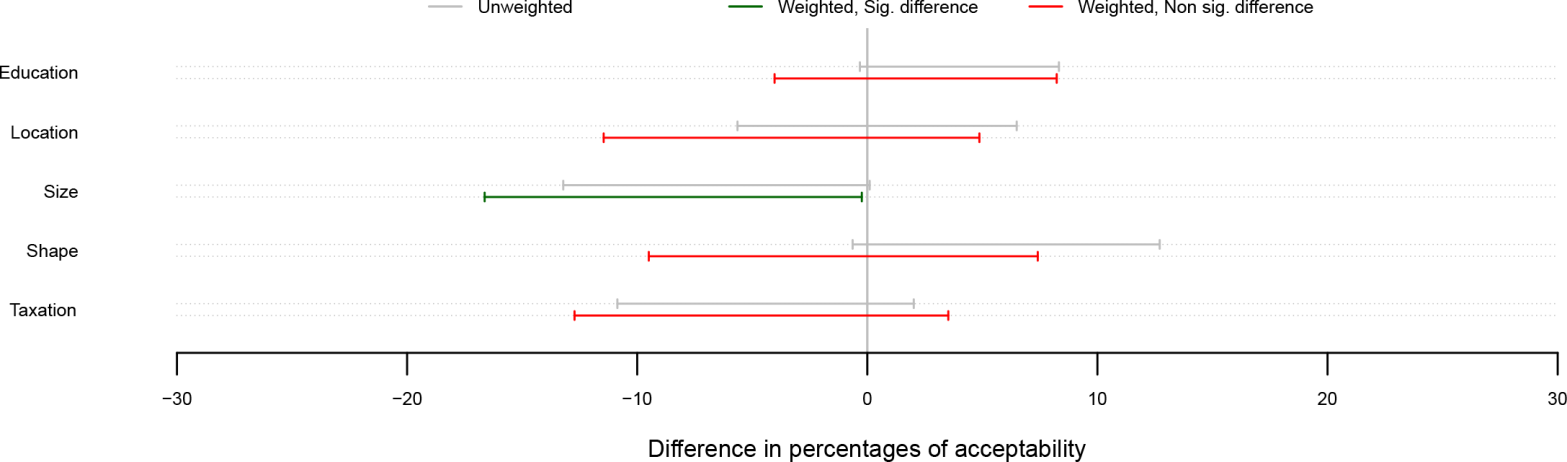
Difference in acceptability (99.807% bootstrap percentile CIs) for each intervention between the US and the UK. Percentages significantly different from 0 appear in green. Percentages not significantly different from 0 appear in red. As 35 comparisons are performed in the analysis corresponding to Figs 2, 3 and 4, a correction for multiple testing which takes into account the dependencies in the data was used, leading to CI levels of 99.807%.

N.B.: As 35 comparisons are performed in the analysis corresponding to Figs 2, 3 and 4, a correction for multiple testing was used, leading to CI levels of 99.807%.

**Table 3:** Unweighted Percentage of participants (% (n)) rating as acceptable [99.807% bootstrap percentile CIs] each intervention, by country.

	UK Sample (n = 1093)	USA Sample (n = 1082)
**Intervention**		
Education	85.8% (938) [82.5;89.0]	89.8% (972) [87.0;92.5]
Location	69.1% (755) [64.7;73.4]	69.4% (751) [65.1;73.7]
Size	59.9% (655) [55.5;64.4]	53.5% (579) [48.8;58.2]
Shape	52.4% (573) [47.7;57.1]	58.5% (633) [53.6;63.1]
Taxation	46.9% (513) [42.3;51.5]	42.4% (459) [37.9;47.0]

N.B.: As 35 comparisons are performed in the analysis corresponding to Figs 2, 3 and 4, a correction for multiple testing was used, leading to CI levels of 99.807%.

As expected, the effect of weighting is greater for the US than the UK data. In both the UK and USA samples, the proportion of participants rating Location and Education as acceptable was significantly higher than 50%. In the US, Taxation was acceptable to significantly fewer than half the participants, while this proportion was not significantly different from 50% in the UK.

The differences in acceptability comparing every possible combination of interventions within each country are shown in Fig 3. Education is acceptable to most in the UK and USA, followed by a change in Location, both of which are more acceptable than changing the Size and Shape of containers, which in turn are more acceptable than Taxation. These results are similar whether weighted or unweighted data are used for the US and UK participants with the exception of the difference between Shape and Taxation in the UK, which are not significant when using unweighted data.

Levels of acceptability for each of the interventions did not differ significantly between the UK and USA samples, using weighted or unweighted data, with the exception of reducing the portion size which was more acceptable in the UK (Fig 4).

### Research question 2: What is the impact on acceptability of highlighting conscious vs. non-conscious mechanisms?

As can be seen in Tables 4 and 5 the effect of experimental condition was not significant for any intervention at p = .00274 in either of the two samples, thus providing no evidence to suggest that highlighting the conscious or non-conscious processes through which interventions affect behavior impacts on acceptability levels.

**Table 4:** Logistic regression models to assess the impact of highlighting mechanism on acceptability for the five interventions in the USA sample.

	Size *B* (*SE*)	Shape *B* (*SE*)	Location *B* (*SE*)	Taxation *B* (*SE*)	Education *B* (*SE*)
(Intercept)	-0.41(0.29)	0.51(0.30)	0.92(0.32)***	-0.13(0.29)	1.55(0.38)***
Conscious condition	0.13(0.15)	-0.16(0.15)	-0.25(0.16)	-0.04(0.15)	0.16(0.27)
Non-conscious condition	0.21(0.15)	0.27(0.16)	0.14(0.17)	-0.02(0.15)	-0.35(0.25)
Gender (F)	1.22(0.50)	0.98(0.57)	0.21(0.53)	-0.60(0.49)	0.67(0.71)
Age [25;30[	0.43(0.45)	0.25(0.48)	0.22(0.51)	0.27(0.45)	1.24(0.81)
Age [30;40[	-0.68(0.50)	-1.07(0.48)	-1.12(0.48)	-0.82(0.50)	0.40(0.64)
Age [40;Inf[	0.15(0.48)	-1.03(0.49)	-0.90(0.49)	0.61(0.49)	-0.25(0.59)
Income [25K,50K[	0.66(0.44)	0.08(0.46)	-0.51(0.46)	-0.21(0.44)	2.01(1.07)
Income [50K,Inf[	0.99(0.41)	0.33(0.43)	0.35(0.46)	0.19(0.40)	0.77(0.59)

N.B.: *** = p < 0.0027358

The control condition taken as reference. To take into account the non-representativeness of the MTurk sample, we control for variables explaining the difference between the MTurk and USA populations. We chose age (4 levels, with [18;25 [y.o. as base), gender (2 levels), and income (3 levels, with [0;25K [as base) and considered all possible interactions between our control variables (i.e., 4x2x3 groups) but only report here their main effects.

**Table 5:** Logistic regression models to assess the impact of highlighting mechanism on acceptability for the five interventions in the UK sample.

	Size *B* (*SE*)	Shape *B* (*SE*)	Location *B* (*SE*)	Taxation *B* (*SE*)	Education *B* (*SE*)
(Intercept)	0.65(0.62)	0.57(0.62)	1.05(0.67)	-0.19(0.59)	1.04(0.67)
Conscious condition	0.06(0.16)	0.24(0.15)	0.05(0.16)	-0.31(0.16)	0.16(0.22)
Non-conscious condition	0.22(0.16)	0.26(0.15)	0.35(0.17)	-0.21(0.16)	-0.05(0.22)
Gender (F)	-1.24(0.74)	-0.39(0.74)	-1.32(0.78)	-1.26(0.80)	0.25(0.84)
Age [30;40[	-1.54(0.79)	-1.79(0.81)	-0.67(0.82)	-2.52(1.18)	0.61(0.92)
Age [40;50[	-0.74(0.69)	-0.32(0.70)	-0.28(0.76)	0.37(0.67)	0.81(0.82)
Age [50;65[	-0.89(0.66)	-1.31(0.66)	-0.74(0.71)	-0.56(0.64)	0.26(0.73)
Age [65;+[	-0.28(0.70)	-0.63(0.70)	-0.47(0.76)	0.15(0.68)	0.74(0.82)
SES–C1C2	-0.84(0.78)	-0.12(0.80)	-1.25(0.83)	-0.81(0.82)	0.44(0.92)
SES–AB	-0.54(0.78)	-0.28(0.78)	-0.53(0.84)	0.11(0.76)	1.80(1.23)

The control condition taken as reference. To take into account the possible non-representativeness of the UK collected sample, we control for variables potentially explaining the difference between the UK sample and population. We chose age (5 levels, with [18;30 [y.o. as base), gender (2 levels), and SES (3 levels, with `DE’ as base) and considered all possible interactions between our control variables (i.e., 5x2x3 groups) but only report here their main effects.

As an exploratory analysis we also tested the effect of ‘conscious versus non-consciousness’. We were not able to reject the hypothesis that consciousness and non-consciousness have the same effect on acceptability for each outcome, at the 5% global type 1 error level.

### Research question 3: What are the independent predictors of acceptability?

Results for both the UK and USA samples are summarised in Tables 6 and 7.

**Table 6:** Logistic regression models predicting acceptability (Yes/NO) for the five interventions in the USA sample.

	Size *B* (*SE*)	Shape *B* (*SE*)	Location *B* (*SE*)	Taxation *B* (*SE*)	Education *B* (*SE*)
(Intercept)	-0.56(0.45)	1.14(0.45)	1.64(0.44)***	-0.76(0.44)	2.72(0.60)***
Conscious condition	0.11(0.22)	-0.32(0.20)	-0.47(0.21)	-0.01(0.21)	-0.03(0.33)
Non-conscious condition	-0.09(0.22)	0.01(0.21)	-0.02(0.21)	0.07(0.21)	-0.14(0.32)
Gender (F)	1.42(0.66)	0.89(0.68)	0.27(0.65)	-1.14(0.66)	0.13(0.87)
Age [25;30[	0.67(0.63)	0.92(0.61)	0.55(0.61)	0.19(0.62)	0.94(0.96)
Age [30;40[	-0.50(0.65)	-0.89(0.66)	-0.52(0.59)	-0.32(0.67)	1.53(0.93)
Age [40;Inf[	0.13(0.71)	-1.23(0.68)	-0.86(0.65)	0.65(0.68)	-0.76(0.84)
Income [25K,50K[	0.74(0.66)	-0.01(0.60)	-0.71(0.59)	-0.58(0.58)	2.13(1.22)
Income [50K,Inf[	0.99(0.58)	0.73(0.58)	0.84(0.57)	0.19(0.54)	0.49(0.77)
Educ. medium	-0.06(0.28)	-0.50(0.27)	-0.53(0.28)	-0.01(0.29)	-0.21(0.41)
Educ. High	0.17(0.28)	-0.18(0.27)	-0.28(0.27)	0.31(0.29)	0.53(0.40)
Ethnic (Latino)	0.84(0.41)	-0.04(0.35)	0.67(0.38)	0.40(0.36)	-0.54(0.53)
Race (non-White)	0.26(0.24)	-0.62(0.24)	-0.47(0.23)	0.18(0.24)	-0.07(0.38)
log BMI	0.01(0.09)	0.17(0.10)	0.06(0.10)	-0.21(0.09)	0.05(0.16)
Political Orientation	-0.10(0.09)	-0.26(0.09)***	-0.22(0.09)	-0.31(0.09)***	-0.68(0.15)***
Trust in Government	0.18(0.10)	0.09(0.09)	-0.05(0.09)	0.07(0.09)	-0.22(0.14)
Situationism	0.13(0.10)	0.05(0.09)	0.10(0.09)	0.20(0.10)	0.07(0.15)
Dispositionism	-0.02(0.10)	0.32(0.10)***	0.20(0.09)	-0.07(0.10)	0.35(0.15)
Help	0.11(0.10)	-0.10(0.09)	0.09(0.09)	0.23(0.10)	0.20(0.16)
Overweight willpower	0.09(0.10)	-0.09(0.10)	0.05(0.10)	-0.01(0.10)	0.01(0.15)
Overweight environment	0.53(0.11)***	0.40(0.10)***	0.44(0.09)***	0.40(0.10)***	0.30(0.14)
Effectiveness	1.95(0.12)***	1.93(0.12)***	1.68(0.12)***	1.93(0.12)***	1.69(0.16)***

N.B.: *** = p<0.005.

The control condition was taken as reference. The assessed racial group categories (Asian, Black/African American, American Indian/Alaska Native, Native Hawaiian/other Pacific islander, multiracial/other) have been collapsed into the category “non-White”. “Ed. College graduate”–college graduate or more was the highest level of education achieved. Gender: men taken as the reference category “effectiveness”–the degree to which each intervention was perceived as effective; “overweight environment”–the belief that the environment is responsible for overweight; “overweight willpower”–the belief that lack of willpower is responsible for overweight.

To take into account the non-representativeness of the MTurk sample, we control for variables explaining the difference between the MTurk and USA populations. We chose age (4 levels, with [18;25[years of age as base), gender (2 levels), and income (3 levels, with [0;25K [as base) and considered all possible interactions between our control variables (i.e., 4x2x3 groups) but only report here their main effects.

All continuous predictors (i.e., log BMI, Political Orientation, Trust in Government, Situationism, Dispositionism, Help, Overweight willpower, Overweight environment, Effectiveness) were standardized.

**Table 7:** Logistic regression models predicting acceptability (Yes/NO) for the five interventions in the UK sample.

	Size *B* (*SE*)	Shape *B* (*SE*)	Location *B* (*SE*)	Taxation *B* (*SE*)	Education *B* (*SE*)
(Intercept)	-0.11(0.90)	0.08(0.85)	1.55(0.86)	-0.78(0.86)	0.62(0.95)
Conscious condition	0.32(0.22)	0.29(0.19)	0.26(0.22)	-0.16(0.23)	-0.14(0.30)
Non-conscious condition	-0.16(0.23)	-0.20(0.20)	0.18(0.23)	-0.21(0.23)	-0.56(0.30)
Gender (F)	0.75(1.04)	0.06(0.99)	-1.39(0.98)	-1.14(1.07)	1.98(1.11)
Age [30;40[	-0.68(1.08)	-1.67(1.01)	-1.27(1.04)	-2.31(1.37)	1.37(1.22)
Age [40;50[	0.28(1.00)	0.42(0.93)	-0.49(0.94)	0.85(1.00)	2.22(1.12)
Age [50;65[	0.45(0.95)	-0.57(0.89)	-0.83(0.89)	-0.29(0.91)	1.46(1.01)
Age [65;+[	0.77(1.03)	-0.52(0.94)	-0.83(0.96)	0.46(0.97)	2.64(1.11)
SES–C1C2	0.49(1.10)	0.19(1.02)	-1.92(1.07)	-0.73(1.14)	0.64(1.17)
SES–AB	-0.78(1.08)	-0.77(1.02)	-0.65(1.05)	-0.44(1.07)	3.10(1.53)
Edu. medium-low	0.25(0.30)	0.40(0.26)	0.55(0.29)	0.05(0.30)	0.48(0.37)
Edu. medium-high	-0.20(0.30)	0.27(0.27)	0.44(0.30)	0.40(0.31)	0.42(0.38)
Edu. High	0.25(0.28)	0.62(0.24)	0.36(0.27)	0.67(0.28)	0.86(0.36)
Ethnic (non-White)	1.03(0.41)	-0.14(0.37)	0.72(0.44)	0.09(0.41)	0.20(0.57)
log BMI	0.04(0.09)	0.04(0.09)	0.03(0.09)	0.05(0.11)	-0.08(0.12)
Political Orientation	-0.08(0.10)	-0.09(0.09)	-0.06(0.10)	-0.02(0.10)	-0.12(0.13)
Trust in Government	0.06(0.10)	0.18(0.09)	-0.06(0.10)	0.07(0.10)	-0.38(0.14)
Situationism	-0.06(0.10)	0.00(0.09)	-0.16(0.10)	-0.10(0.10)	-0.21(0.13)
Dispositionism	0.23(0.10)	0.19(0.09)	0.19(0.10)	0.02(0.10)	0.32(0.12)
Help	-0.15(0.11)	0.05(0.09)	0.00(0.10)	-0.06(0.11)	0.09(0.14)
Overweight willpower	-0.10(0.10)	0.08(0.09)	0.14(0.10)	0.04(0.11)	0.18(0.12)
Overweight environment	0.40(0.11)***	0.09(0.09)	0.47(0.10)***	0.25(0.11)	0.55(0.12)***
Effectiveness	2.14(0.13)***	1.64(0.11)***	1.81(0.13)***	2.18(0.13)***	1.83(0.16)***

N.B.:*** = p<0.005.

The control condition was taken as reference. The assessed racial group categories (Asian, Black/African American, American Indian/Alaska Native, Native Hawaiian/other Pacific islander, multiracial/other) have been collapsed into the category “non-White”. “Ed. College graduate”–college graduate or more was the highest level of education achieved; BMI stands for body-mass index. Gender: men taken as the reference category “effectiveness”–the degree to which each intervention was perceived as effective; “overweight environment”–the belief that the environment is responsible for overweight; “overweight willpower”–the belief that lack of willpower is responsible for overweight.

To take into account the possible non-representativeness of the UK collected sample, we control for variables potentially explaining the difference between the UK sample and population. We chose age (5 levels, with [18;30[y.o. as base), gender (2 levels), and SES (3 levels, with `DE’ as base) and considered all possible interactions between our control variables (i.e., 5x2x3 groups) but only report here their main effects.

All continuous predictors (i.e., log BMI, Political Orientation, Trust in Government, Situationism, Dispositionism, Help, Overweight willpower, Overweight environment, Effectiveness) were standardized.

#### UK sample

Perceived effectiveness was the strongest predictor for each intervention. B values ranged from B = 1.64 for Shape to 2.18 for Taxation. Belief that the environment is responsible for obesity was a significant predictor for Size (B = .40; SE = .11), Location (B = .47; SE = .10), and education (B = .55; SE = .12). No other variable reached significance. The robust estimator replicated the results obtained using the classical logistic regression.

#### USA sample

Perceived effectiveness was again the strongest predictor of acceptability for each intervention with B values ranging from B = 1.68 for Location to 1.95 for Size. Belief that the environment is responsible for obesity was a significant predictor for all interventions except Education. B values ranged from B = .40 for Taxation and shape to.53 for Size. In line with the UK analysis, using the robust estimator led to the same findings as the classical logistic regression.

## Discussion

Levels of acceptability for four of the five interventions did not differ significantly between the UK and USA samples. Reducing portion Size was less accepted in the USA than the UK sample. Overall, acceptability was lowest for the intervention involving a high-level of intrusion, i.e. Taxation, and highest for the intervention presenting a low level of intrusion, i.e. Education. Contrary to prediction, there was no evidence that emphasizing the non-conscious route through which nudge interventions are expected to work reduces their acceptability. Perceived effectiveness of interventions predicted acceptability for all interventions across both samples. Believing that the environment is responsible for obesity was also a reliable predictor of acceptability for most policies.

The present study replicates previous research with regard to the acceptability of traditional interventions to improve population health, with Education attracting the most and Taxation the least support [12]. As noted previously, nudging to improve health (and other outcomes) has been criticised by ethicists and others for potentially undermining autonomy and being coercive [10, 11, 15]. Here we report findings suggesting that the majority of the public may find at least one type of nudging intervention acceptable, changing the shelf Location of sugar-sweetened beverages containers. The difference between the UK and USA samples in acceptability of one nudge—reducing portion size–may reflect the negative response to an attempt in New York to cap the size of sugar sweetened beverage sales [37]. Public acceptability appears to be conditional on the nature of the nudge as well as other considerations. Evidence to support this comes from experimental studies in health and other contexts that highlight the importance of people valuing the motivations of those applying nudges as well as trusting them [38, 39].

We found no evidence to support the study hypothesis, based on findings from just one previous study [16]: highlighting the non-conscious nature of the mechanisms through which nudge interventions are expected to influence behavior did not reduce intervention acceptability. There are several possible explanations for differences in findings between the present study and those of Felsen and colleagues. First, these may reflect differences between the two studies in the words used to describe non-conscious processes. Felsen et al., used the words “subconsciously driven bias” to describe interventions that would affect behavior through non-conscious processes. In the present study the wording used was “people will not be conscious (i.e. not aware)” of the way in which the intervention would affect their behavior. We did not use the same wording as Felsen et al., in order to avoid the negative connotations of the terms “subconscious” and “*bias*”. It is therefore possible that the negative effects reported by these authors were primed by these terms rather than to the concept of non-consciousness *per se*. Second, the present study tested the hypothesis that information about mechanism influences acceptability whilst the intervention itself is kept constant across experimental conditions. By contrast, in the study reported by Felsen et al., interventions differed by experimental condition. For example in the “eating scenario” participants in the “subconscious” condition were presented with an intervention altering the placement of products, whereas participants in the “conscious” condition were presented with a labelling intervention. Therefore differences between groups could be attributed to the manipulation, the nature of the intervention in each condition, or a combination of the two. Third, in the present study acceptability was assessed directly. This was not the case for Felsen et al.’s study, in which a proxy measure was used: whether or not participants would prefer working for a company that used the intervention.

With regard to independent predictors of acceptability, across all interventions, the strongest predictor of acceptability was perceived effectiveness of interventions. This concurs with other evidence concerning other interventions and behaviors [17, 40–42]. For example, in a discrete-choice experiment, Pechey and colleagues manipulated the expected effectiveness of various interventions designed to decrease alcohol consumption [17]. Results revealed that both the type of intervention and its anticipated effectiveness influenced public acceptability.

In the absence of specific information regarding the expected effectiveness of interventions, participants’ expectations with regard to effectiveness emerged as a strong predictor of intervention acceptability. However, people often hold inaccurate beliefs about the effectiveness of interventions to change behaviour [12]. Such inaccuracies might reflect a process of cognitive consistency such that less acceptable interventions are seen as less effective. This could explain why less acceptable interventions, such as taxation, are perceived as less effective than they actually are. The belief that the environment is responsible for people being overweight was also a predictor of acceptability for most interventions, across the both the UK and USA samples, in keeping with other evidence [18].

The main strength of the current study is that it is, to our knowledge, the first robust assessment of the public acceptability of nudge interventions to improve population health. It is also the first study to directly compare responses from UK and USA participants. The observation that levels of acceptability did not seem to differ between the two countries for most interventions provides preliminary evidence to “read across” in each country from studies assessing acceptability of government intervention to change behavior to improve population health. A potential limitation of the current study concerns the different sampling methods used to recruit participants in the two countries with resulting sample differences. The USA sample was recruited using the same panel as that used by Felsen and colleagues, for reasons of study comparability. This method was not practicable in the UK. It is also important to note that neither of the two study samples was nationally representative. The UK sample was similar to a nationally representative sample in terms of gender and age but not social grade. The US sample differed from national representativeness in terms of gender, age and social grade, as measured by income. We controlled for demographic characteristics in analyses, and also separately conducted analyses by calculating weights based on census data for relevant demographic characteristics. The conclusions from analyses conducted using unweighted and weighted data were mostly unchanged (two exceptions being the acceptability of changing the Size in the UK and the difference between Taxation and changing the Shape in the UK), providing some assurance that the unrepresentativeness of the study samples does not undermine the study findings. The fact that conclusions remain the same when using unweighted and weighted samples suggests that generally our samples do not deviate markedly from national representativeness, regarding the question of interest. More specifically, the variables thought to have an effect on sampling do not seem to impact on acceptability. In fact, when the impact of these demographic variables on acceptability is assessed in Research question 3, they do not appear as significant predictors. Nonetheless, the possibility remains that other unmeasured factors impacted sampling probability. A further limitation of the present study is the loss of power resulting from dichotomising the primary outcome variable. The possibility remains that alternative hypotheses might have been accepted had the distribution of this variable allowed analyses using more powerful models.

We would also note that data collected using Amazon Mechanical Turk (MTurk) has been found to be of high quality when judged against psychometric criteria [43] and participant attention [44]. With regard to sample characteristics, a recent study, conducted by Berinsky et al., [45] comparing MTurk with more traditional recruitment methods concluded that “*the MTurk sample does not perfectly match the demographic and attitudinal characteristics of the U*.*S*. *population but does not present a wildly distorted view of the U*.*S*. *population either*” (p.361). Weighting data to address this goes some way towards overcoming this limitation. Subsequent studies should build on the current findings using larger and more representative samples. A second limitation of the current study is that we did not assess the degree to which participants thought the interventions were infringing on their autonomy. Again, this could usefully be included in subsequent studies.

A further focus for future studies is consideration of the extent to which the current findings generalise to other interventions and other behaviors. Further research is also needed to unravel the possible causal nature of the observed association between effectiveness of interventions and their public acceptability. While it is plausible that perceived effectiveness of the interventions studied has a direct causal influence on public acceptability, in keeping with other evidence [17, 40], studies are needed to test this hypothesis.

The results presented here have implications for policy. They suggest that, in the context of reducing sugar-sweetened beverages consumption, highlighting the non-conscious routes by which nudge-type interventions have their effects will not reduce public acceptability. In addition, our results suggest that the majority of the public in the UK and the USA are likely to find at least one type of nudge intervention–changing Location of products to reduce their selection and consumption–acceptable. Furthermore the majority of the UK sample finds changing the Size of sugar sweetened beverages containers acceptable. This is particularly pertinent for the UK given that reducing portion sizes has been highlighted as a policy option supported by evidence for the forthcoming Childhood Obesity Strategy for England [46]. Nudge interventions may be developed and implemented by policymakers, but they may also be implemented through voluntary partnerships, and regulatory and quasi–regulatory measures [47, 48]. While policy makers might be reassured about the public acceptability of nudge type interventions in the current context, the results of the present study provide no such reassurance for using Taxation to reduce consumption, consonant with other evidence [12]. Given the potentially large effects on behaviour of pricing interventions further work is justified to consider whether and how public acceptability for such interventions might be increased.

The main predictors of the acceptability of interventions in the current study, perceived effectiveness and attributing being overweight to the environment, might also help policymakers and public health practitioners to frame effective policies to maximise their support by the public [37], support that could even increase public demand for policy change to prevent obesity and other health-related problems [49].

## Supporting Information

S1 Appendix Measures for Predictors of acceptability. (DOCX)

S2 Appendix **Box A.** Descriptions of each of the five interventions by study group.
(DOCX)

S3 Appendix Data—Acceptability USA. Data—Acceptability UK.
(ZIP)
