# Supplementary material for: Public Acceptability in the UK and USA of Nudging to Reduce Obesity: The Example of Reducing Sugar-Sweetened Beverages Consumption
Source: PLoS One. 2016 Jun 8;11(6):e0155995. doi: 10.1371/journal.pone.0155995 (PMC4898693; doi:10.1371/journal.pone.0155995)
Supplement: S1 Appendix — (DOCX) [file pone.0155995.s001.docx]

**Supporting Information S1**

**Measures for predictors of acceptability**

**Perceived effectiveness of intervention (assessed after each intervention)**

“How effective do you think this policy will be?”

**Dispositionism vs. Situationism**

“Your behavior is mainly determined by how you choose to act”;

“Your behavior is mainly determined by the environment around you”;

“Other people’s behavior is mainly determined by how they choose to act”;

“Other people’s behavior is mainly determined by the environment around them”.

**Beliefs regarding the causes of overweight**

“People are overweight because there are so many unhealthy foods around”;

“People are overweight because they lack willpower” (1).

**Trust in government**

“Overall, how confident do you feel in the way the government of the United States / government of the United Kingdom tackles the main problems facing the country?” (2).

**Political orientation**

“In general, how liberal or conservative are you with regard to social, economic, and political issues?”

**Perceived need for help in making healthier choices**

“To what extent do you feel like you could use help making healthier eating choices?” (3).

**Quality-check items** (adapted from previous research) (4)

“You have been to all the countries in the world” (Requires participants to give a negative answer);

“You sleep more than an hour per night” (Requires participants to give a positive answer).
