# Supplementary material for: Public Acceptability in the UK and USA of Nudging to Reduce Obesity: The Example of Reducing Sugar-Sweetened Beverages Consumption
Source: PLoS One. 2016 Jun 8;11(6):e0155995. doi: 10.1371/journal.pone.0155995 (PMC4898693; doi:10.1371/journal.pone.0155995)
Supplement: S2 Appendix — Box A. Descriptions of each of the five interventions by study group. (DOCX) [file pone.0155995.s002.docx]

**S2 Appendix**

**Box A.** Descriptions of each of the five interventions, by study group

| Intervention type: study group | **Size of container**  **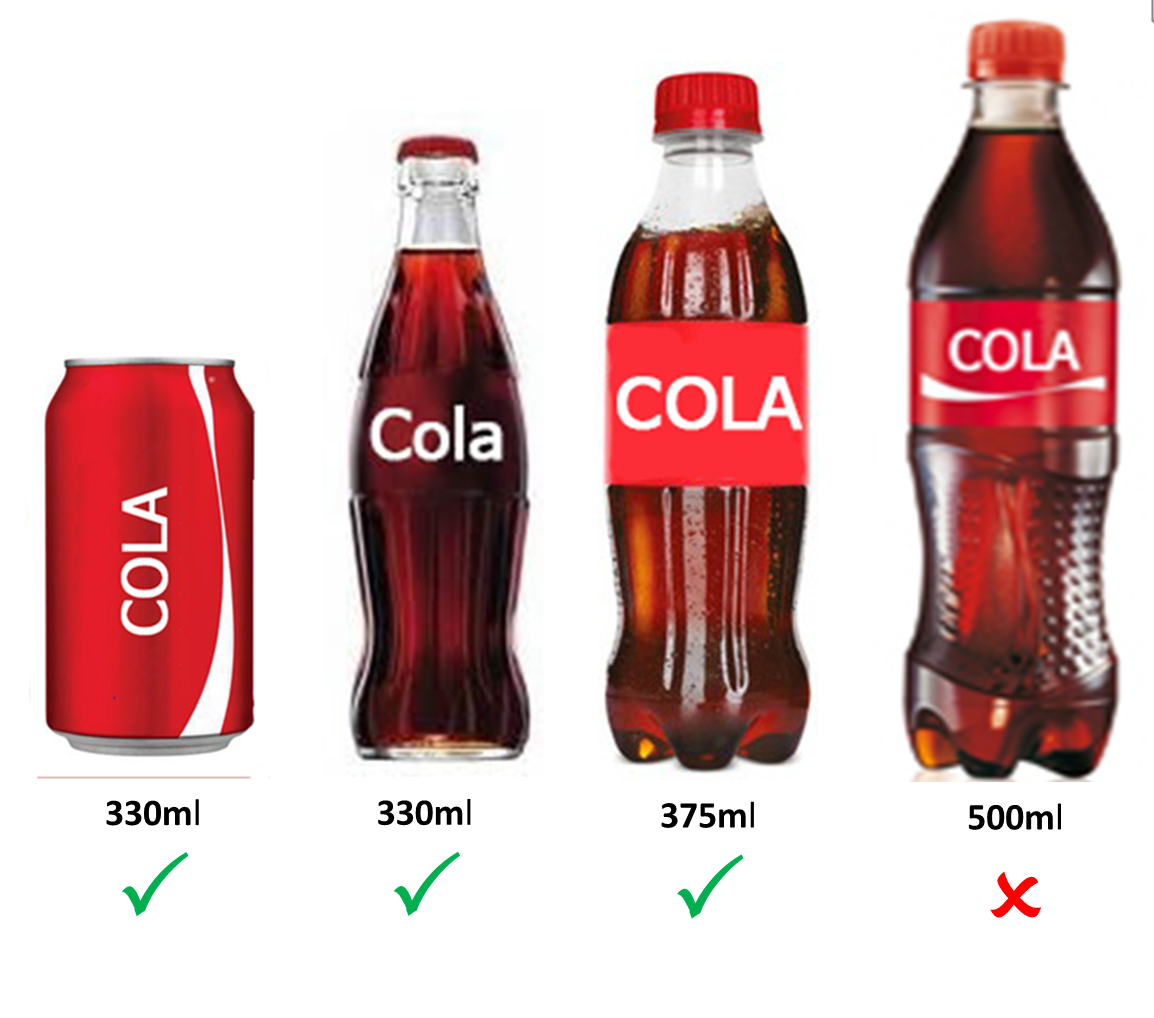**  Participants saw an image with four sugar-sweetened beverages containers and colour coded “tick” and “cancel” signs, which indicated that only small containers will be available. | **Shape of container**  **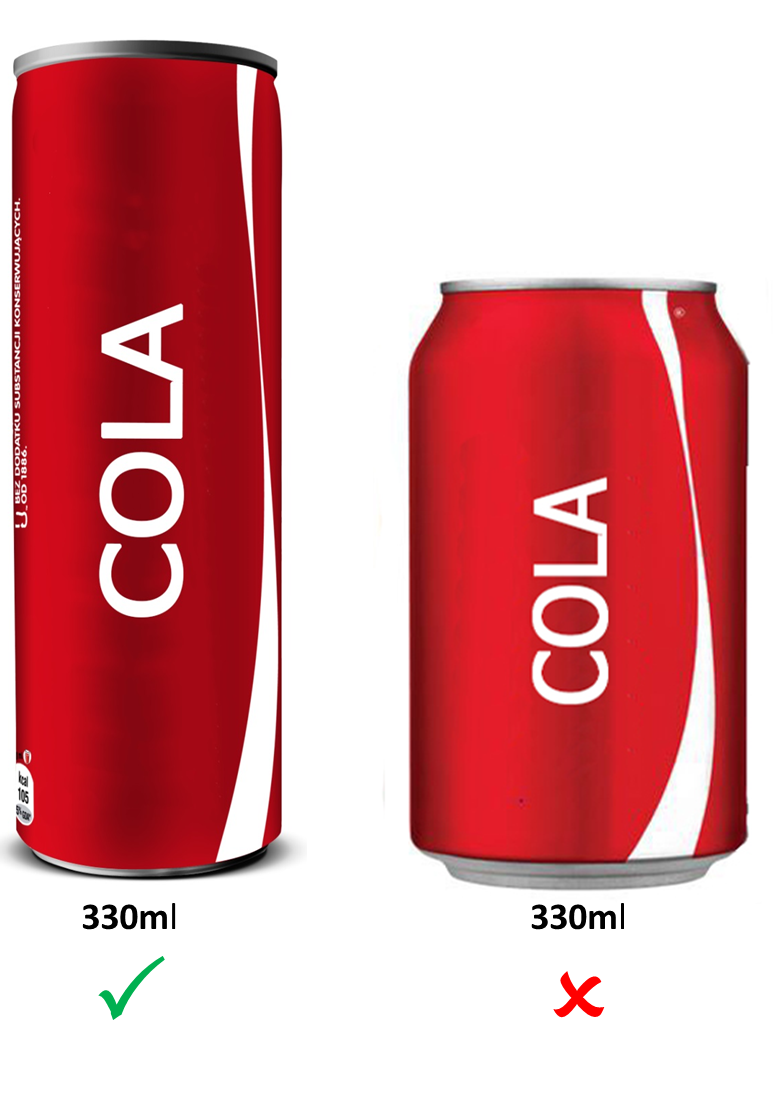**  Participants saw an image with two sugar-sweetened beverages cans and colour coded “tick” and “cancel” signs, which indicated that the tall and narrow can will be available, whereas the shorter and wider can will not. | **Location of sugary drinks**  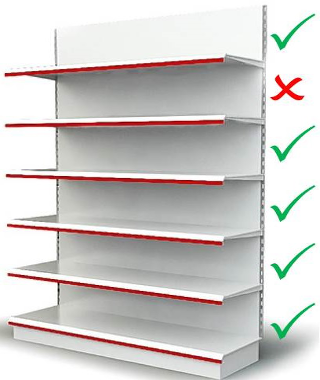  Participants saw an image with several empty shelves and colour coded “tick” and “cancel” signs, which indicated that sugar-sweetened beverages will not be placed at eye-level. | **Increased taxation**  **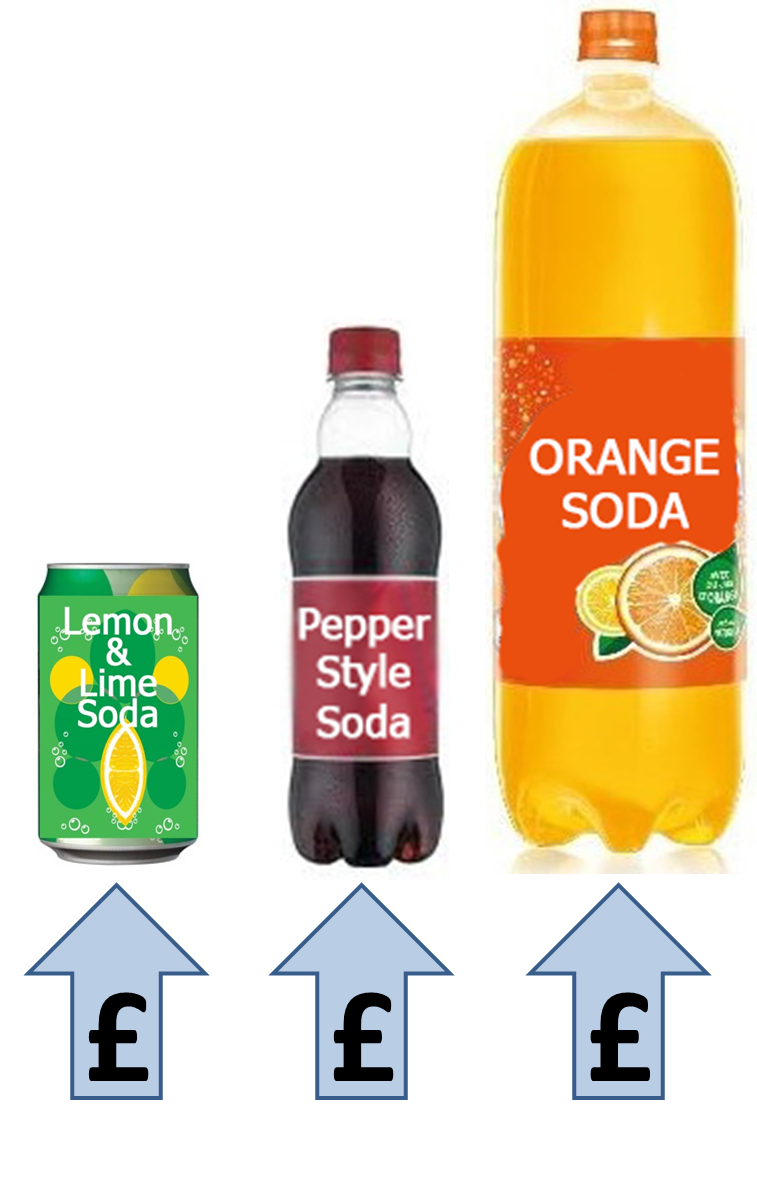**  Participants saw three bottles of sugar-sweetened beverages and arrow marks and currency symbols, which indicated prices will increase. | **Health education**  **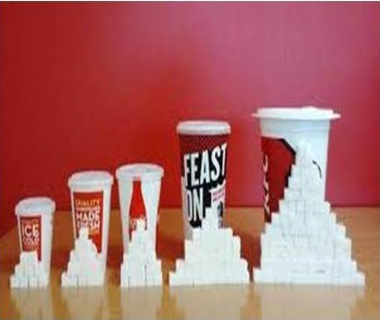**  Participants saw an image of five sugar-sweetened beverages containers and the quantity of sugar they each contain. |
| --- | --- | --- | --- | --- | --- |
| **Conscious** | This new policy would work like this:  -The size of sugary drinks containers (e.g., bottles & cans) will be limited to smaller versions  -Changing the size of containers for sugary drinks means people will tend to drink less  -People will be conscious (i.e. aware) of how this change in container size makes them drink less  -People will still be able to drink as much as they like | This new policy would work like this:  -The shape of sugary drinks containers (e.g., bottles & cans) will be made taller and narrower  -Changing the shape of containers for sugary drinks means people will tend to drink less  -People will be conscious (i.e. aware) of how this change in container shape makes them drink less  -People will still be able to drink as much as they like | This new policy would work like this:  -Sugary drinks will be placed so that they are not at customers' eye-level  -Changing the location of sugary drinks means people will tend to drink less  -People will be conscious (i.e. aware) of how this change in location makes them drink less  -People will still be able to drink as much as they like | This new policy would work like this:  -There will be an extra tax on sugary drinks so they will cost more to buy  -Changing the price of sugary drinks means people will tend to drink less  -People will be conscious (i.e. aware) of how this change in price makes them drink less  -People will still be able to drink as much as they like | This new policy would work like this:  -There will be a health education campaign (e.g. adverts, posters) about the harmful effects of sugary drinks  -Health education on the effects of sugary drinks means people will tend to drink less  -People will be conscious (i.e. aware) of how this education campaign makes them drink less  -People will still be able to drink as much as they like |
| **Non-conscious** | This new policy would work like this:  -The size of sugary drinks containers (e.g., bottles & cans) will be limited to smaller versions  -Changing the size of containers for sugary drinks means people will tend to drink less  -People will not be conscious (i.e. not aware) of how this change in container size makes them drink less  -People will still be able to drink as much as they like | This new policy would work like this:  -The shape of sugary drinks containers (e.g., bottles & cans) will be made taller and narrower  -Changing the shape of containers for sugary drinks means people will tend to drink less  -People will not be conscious (i.e. not aware) of how this change in container shape makes them drink less  -People will still be able to drink as much as they like | This new policy would work like this:  -Sugary drinks will be placed so that they are not at customers' eye-level  -Changing the location of sugary drinks means people will tend to drink less  -People will not be conscious (i.e. not aware) of how this change in location makes them drink less  -People will still be able to drink as much as they like | This new policy would work like this:  -There will be an extra tax on sugary drinks so they will cost more to buy  -Changing the price of sugary drinks means people will tend to drink less  -People will not be conscious (i.e. not aware) of how this change in price makes them drink less  -People will still be able to drink as much as they like | This new policy would work like this:  -There will be a health education campaign (e.g. adverts, posters) about the harmful effects of sugary drinks  -Health education on the effects of sugary drinks means people will tend to drink less  -People will not be conscious (i.e. not aware) of how this education campaign makes them drink less  -People will still be able to drink as much as they like |
| **Control group** | This new policy would work like this:  -The size of sugary drinks containers (e.g., bottles & cans) will be limited to smaller versions  -Changing the size of containers for sugary drinks means people will tend to drink less  -People will still be able to drink as much as they like | This new policy would work like this:  -The shape of sugary drinks containers (e.g., bottles & cans) will be made taller and narrower  -Changing the shape of containers for sugary drinks means people will tend to drink less  -People will still be able to drink as much as they like | This new policy would work like this:  -Sugary drinks will be placed so that they are not at customers' eye-level  -Changing the location of sugary drinks means people will tend to drink less  -People will still be able to drink as much as they like | This new policy would work like this:  -There will be an extra tax on sugary drinks so they will cost more to buy  -Changing the price of sugary drinks means people will tend to drink less  -People will still be able to drink as much as they like | This new policy would work like this:  -There will be a health education campaign (e.g. adverts, posters) about the harmful effects of sugary drinks  -Health education on the effects of sugary drinks means people will tend to drink less  -People will still be able to drink as much as they like |
